# Glucagon-Like Peptide-1 Receptor Agonist Use and Renal Impairment: A Retrospective Analysis of an Electronic Health Records Database in the U.S. Population

**DOI:** 10.1007/s13300-018-0377-5

**Published:** 2018-02-19

**Authors:** Kristina S. Boye, Fady T. Botros, Axel Haupt, Brad Woodward, Maureen J. Lage

**Affiliations:** 1 Global Patient Outcomes and Real World Evidence, Eli Lilly and Company, Lilly Corporate Center, Indianapolis, IN 46285 USA; 2 Diabetes, Eli Lilly and Company, Lilly Corporate Center, Indianapolis, IN 46285 USA; 3 Early Phase Clinical Research—Diabetes and Complications, Eli Lilly and Company, Lilly Corporate Center, Indianapolis, IN 46285 USA; 4 HealthMetrics Outcomes Research, 27576 River Reach Drive, Bonita Springs, FL 34134 USA

**Keywords:** Estimated glomerular filtration rate, Glucagon-like peptide-1 receptor agonists, Glycated hemoglobin, Renal impairment

## Abstract

**Introduction:**

The study characterizes the use of glucagon-like peptide-1 receptor agonists (GLP-1 RAs) in patients with type 2 diabetes (T2D) with and without renal impairment and examines the effects of such use on the clinical outcomes of estimated glomerular filtration rate (eGFR) and glycated hemoglobin (A1c).

**Methods:**

Data from the Practice Fusion electronic health records database from 1 January 2012 through 30 April 2015 were used. Adults with T2D who received serum creatinine laboratory tests and initiated therapy with a GLP-1 RA (*N* = 3225) or other glucose-lowering agent (GLA) (*N* = 37,074) were included in the analysis. The GLP-1 RA cohort was matched to cohorts initiating therapy any other GLA, and multivariable analyses examined the association between GLP-1 RA use and changes in eGFR or A1c at 1 year after therapy initiation.

**Results:**

In this study, only 5.7% of patients with an eGFR of < 30 and ≥ 15 mL/min/1.73 m^2^ and 3.6% of patients with an eGFR of  < 15 mL/min/1.73 m^2^ initiated therapy with a GLP-1 RA. Compared to other GLAs, at 1-year after initiation of therapy the use of a GLP-1 RA was associated with a significantly smaller decline in eGFR (− 0.80 vs. − 1.03 mL/min/1.73 m^2^; *P* = 0.0005), a significantly smaller likelihood of having a ≥ 30% reduction in eGFR (2.19 vs. 3.14%; *P* < 0.0001), and a significantly larger reduction in A1c (− 0.48 vs. − 0.43; *P* = 0.0064).

**Conclusion:**

In clinical practice, the use of GLP-1 RAs in patients with a higher degree of renal impairment disease was limited. Compared to other GLAs, the use of GLP-1 RAs was associated with a significantly smaller decline in eGFR and a larger reduction in A1c over the 1 year following therapy initiation.

**Funding:**

Eli Lilly and Company.

## Introduction

Chronic kidney disease (CKD) is a common type 2 diabetes (T2D) complication that is associated with an increased risk of adverse outcomes [1]. For example, the U.S. Renal Data System attributed diabetes as the primary cause of end-stage renal disease (ESRD) in 44.3% of incident dialysis patients in 2011 [2], and patients with comorbid T2D and CKD have been reported to experience increased rates of cardiovascular morbidity and mortality [3]. The presence of CKD also increases the complexity of T2D treatment, since the pharmacokinetic aspects of drugs cleared by the kidney can be influenced by renal impairment [4]. Treatment options are also limited for these patients, with biguanides, alpha-glucosidase inhibitors, and sodium–glucose cotransporter 2 (SGLT2) inhibitors not usable in patients with ESRD and T2D, and thiazelidinediones not usable in patients with ESRD and diabetes with cardiac disease [5]. In addition, the incidence of hypoglycemia is increased in patients with both diabetes and CKD [6].

Animal studies have indicated a renoprotective effect with the use of glucagon-like peptide-1 receptor agonist (GLP-1 RA) medications [7, 8]. Clinical studies have also shown GLP-1 RAs to be associated with decreased development and progression of nephropathy [9, 10], primarily driven by lower rates of new-onset persistent macroalbuminuria [11]. However, the U.S. Food Drug Administration (FDA) label for GLP-1 RA exenatide states that the drug “should not be used in patients with severe renal impairment or end-stage renal disease and should be used with caution in patients with renal transplantations” [12], and the FDA’s label for lixisenatide recommends monitoring of renal function when initiating or escalating doses of the drug and states that the drug “is not recommended in patients with end stage renal disease” [13].

To investigate how GLP-1 RAs are being prescribed in real-world settings, we have examined the frequency of initiation on drugs in this class across various estimated glomerular filtration rate (eGFR) categories in patients with T2D. In addition, given the burden of CKD among patients with T2D and the difficulties in treating such patients, in the current study we also explored the association between initiation of therapy with a GLP-1 RA and changes in glycated hemoglobin (A1c) and kidney function, as measured by the eGFR.

## Methods

### Data

The Practice Fusion electronic health record database furnished the study data, which covered the time period from 1 January 2012 through 30 April 2016. Practice Fusion’s web-based electronic health records (EHR) system contains data input by over 150,000 medical professionals primarily working in small, ambulatory practices and primary care practices and treating over 50 million patients in all 50 states [14]. In particular, Practice Fusion provided recent records on patient characteristics, diagnoses, medications prescribed, laboratory test results, and observational data, such as weight and blood pressure. All records were de-identified and fully compliant with Health Insurance Portability and Accountability Act regulations. The research does not contain any studies with human participants or animals performed by any of the authors.

### Inclusion and Exclusion Criteria

This study focused exclusively on patients identified as having T2D over the time period from 1 January 2013 through 1 May 2015 (i.e., the identification window), based upon a validated algorithm designed for EHR data [15]. Specifically, all patients who received at least one diagnosis of diabetes (International Classification of Diseases, Ninth Revision, Clinical Modification [ICD-9-CM], code 250.xx) were initially considered. Patients were excluded if they received any of the following: (1) more diagnoses of type 1 diabetes (T1D; ICD-9-CM codes 250.x1, 250.x3) than of T2D (ICD-9-CM codes of 250.x0, 250.x2) in addition to a prescription for glucagon; (2) more diagnoses of T1D than of T2D with no record of receipt of a GLA other than metformin; (3) a negative laboratory test result for c-peptide or a positive test result for diabetes autoantibody; or (4) a prescription for a urine acetone test strip.

**Fig. 1:**
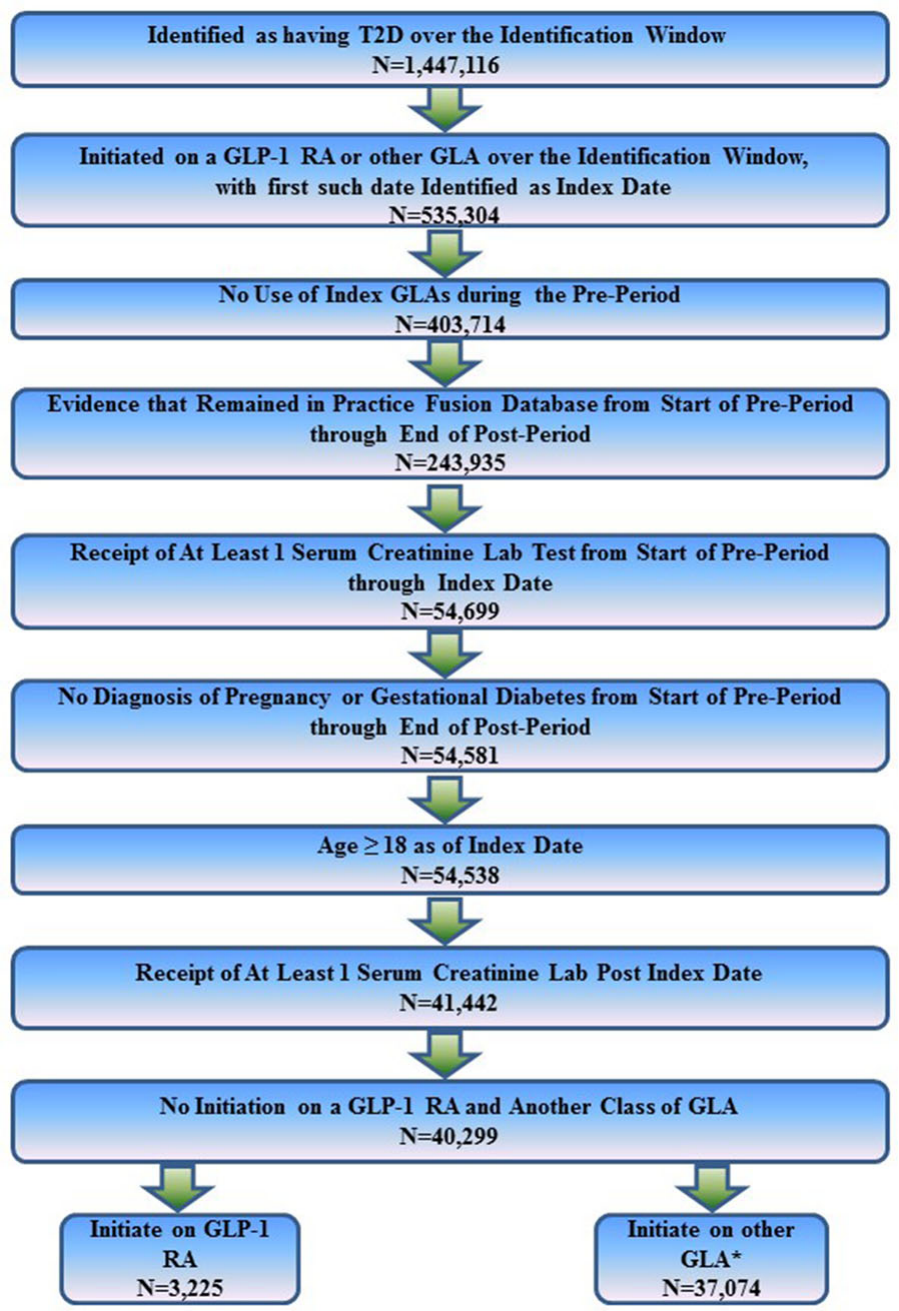
Inclusion–exclusion criteria and sample size of study.* GLA* Glucose-lowering agent,* GLP-1 RA* glucagon-like peptide-1 receptor agonist,* T2D* type 2 diabetes

Patients were included in the analysis if they initiated therapy with a glucose-lowering agent (GLA) during the identification window, with the first such date identified as the index date. Patients were excluded if they: (1) received a prescription for their index class of medication during the 1-year prior to the index date (i.e., during the pre-period); (2) received a diagnosis of pregnancy at any time from the start of the pre-period through the 1 year following the index date (i.e., the post-period); (3) were younger than 18 years as of the index date; or (4) appeared to have dropped out of the database at any time from the start of the pre-period through the end of the post-period. Patients were also required to have at least two recorded serum creatinine laboratory test results, with the first such test occurring some time after the start of the pre-period through to the index date and the second serum creatinine test recorded in the post-period. The eGFR was estimated using the Chronic Kidney Disease Epidemiology Collaboration (CKD-EPI) equation [16]. Patients were classified based on their eGFR categories (eGFR ≥ 90 mL/min/1.73 m^2^; eGFR < 90 and ≥ 60 mL/min/1.73 m^2^; eGFR < 60 and ≥ 45 mL/min/1.73 m^2^; eGFR < 45 and ≥ 30 mL/min/1.73 m^2^; eGFR < 30 and ≥ 15 mL/min/1.73 m^2^; or eGFR < 15 mL/min/1.73 m^2^) [17]. These criteria resulted in a sample of 40,299 patients—3225 of whom initiated therapy with a GLP-1 RA and 37,074 of whom initiated therapy with an alternative class of GLA. Figure 1 illustrates how each of the inclusion and exclusion criteria affected sample size.

### Statistical Methods

The GLP-1 RA and other GLA cohorts were matched using Mahalanobis matching, with calipers defined by the propensity score [18]. Multivariable analyses were then used to examine differences between patients who were treated with a GLP-1 RA and those treated with an alternative GLA. The analyses examined changes in eGFR over the study period as well as the likelihood of having a ≥ 30% reduction in eGFR. A similar analysis was conducted for the subset of patients who also had a A1c laboratory test recorded during both the pre- and post-period, with the multivariable analysis examining the change in A1c. All multivariable analyses were controlled for patient demographics, observational patient characteristics, general health and comorbidities, prior medication use and resource utilization, as well as index A1c and eGFR values. Patient characteristics included age, sex, race, ethnicity, region of residence, and smoking status, while observational characteristics included body mass index and blood pressure. General health was proxied by the Charlson Comorbidity Index (CCI) [19, 20]. Diabetes-specific health was measured by the Diabetes Complications Severity Index (DCSI) [21]. Anxiety and depression were also studied, as these comorbidities have been shown to be both prevalent in patients with diabetes [22, 23] and associated with poorer patient outcomes, and as they are not included in either the CCI or the DCSI. Prior medication use focused on which classes of GLA drugs were prescribed in the pre-period, the number of classes of anti-hypertensive drugs prescribed, and whether any of the following medications were prescribed: angiotensin-converting enzyme inhibitors, angiotensin receptor blockers, hypertension fixed-dose combination therapy, non-steroidal anti-inflammatory drugs (NSAIDs), aspirin, statins, and ezetimibe. Prior resource utilization captured the number of visits to a cardiologist or endocrinologist.

Given the results of the multivariable analyses, differences in continuous outcomes were examined using* t* statistics, with a *P* value of < 0.05 considered, a priori, to be statistically significant. When examining the probability of having a ≥ 30% reduction in eGFR, odds ratios (ORs) and 95% confidence intervals (CIs) were recorded. All analyses were conducted using SAS version 9.4 (SAS Institute, Cary, NC, USA).

## Results

Table 1 presents unadjusted descriptive statistics both before and after matching and reveals significant differences between patients who initiated therapy with a GLP-1 RA compared to those who initiated therapy with an alternative class of GLA. Patients who initiated treatment with a GLP-1 RA were significantly younger (58.5 vs. 63.0 years; *P* < 0.001) and more likely to be identified as white (57.27 vs. 48.98%; *P* < 0.001) and non-Hispanic (73.21 vs. 69.43%; *P* < 0.001). These patients were also less likely to be identified as underweight/normal weight (3.97 vs. 10.96%) or overweight (14.36 vs. 25.48%) and more likely to be identified as class II obese (21.86 vs. 15.71%) or class III obese (26.70 vs. 14.43%). Examination of differences in pre-period general health, comorbidities, GLA use, other medication use, and visits to specialists generally suggested that patients who initiated therapy with a GLP-1 RA were in poorer health than patients who initiated therapy with other GLAs. For example, patients who started taking a GLP-1 RA were found to have a significantly higher CCI score (1.53 vs. 1.36; *P* < 0.001), and they were more likely to be diagnosed with comorbid depression (11.47 vs. 8.36%; *P* < 0.001). Furthermore, these patients were significantly more likely to have received a prescription for hypertensive drugs, NSAIDs, statins, aspirin, or ezetimibe. Patients who initiated therapy on a GLP-1 RA compared to alternative GLAs were prescribed more GLAs in the pre-period and had a significantly higher mean index A1c (8.47 vs 7.77%; *P* < 0.001).

**Table 1:** Patient characteristics pre- and post matching

Patient characteristics pre- and post matching	Prior to matching			Post matching		
	GLP-1 RA (*N* = 3225)	Other GLA (*N* = 37,074)	*P* value	GLP-1 RA (*N* = 2966)	Other GLA (*N* = 2966)	*P* value
**Demographics and Smoking Status**						
Age (years)	58.49 ± 11.77	62.99 ± 12.55	< 0.001	58.84 ± 11.68	59.07 ± 12.43	0.475
Sex			0.167			0.714
Male	1524 (47.26)	18,161 (48.99)		1408 (47.47)	1430 (48.21)	
Female	1700 (52.71)	18,900 (50.98)		1557 (52.49)	1534 (51.72)	
Unknown	1 (0.03)	13 (0.04)		1 (0.03)	2 (0.07)	
Race			< 0.001			0.731
African-American	488 (15.13)	5638 (15.21)		450 (15.17)	437 (14.73)	
White	1847 (57.27)	18,159 (48.98)		1679 (56.61)	1720 (57.99)	
Other	380 (11.78)	6134 (16.55)		364 (12.27)	359 (12.10)	
Unknown	510 (15.81)	7143 (19.27)		473 (15.95)	450 (15.17)	
Ethnicity			< 0.001			0.951
Hispanic	477 (14.79)	6175 (16.66)		433 (14.60)	434 (14.63)	
Non-Hispanic	2361 (73.21)	25,739 (69.43)		2165 (72.99)	2172 (73.23)	
Unknown	387 (12.00)	5160 (13.92)		368 (12.41)	360 (12.14)	
Region			< 0.001			0.478
Northeast	453 (14.05)	6015 (16.22)		426 (14.36)	412 (13.89)	
Midwest	394 (12.22)	3635 (9.80)		365 (12.31)	353 (11.90)	
South	1681 (52.12)	20,071 (54.14)		1548 (52.19)	1560 (52.60)	
West	688 (21.33)	7082 (19.10)		618 (20.84)	623 (21.00)	
Other	9 (0.28)	271 (0.73)		9 (0.30)	18 (0.61)	
Smoking status			0.123			0.074
Never smoker	1516 (47.01)	17,028 (45.93)		1388 (46.80)	1330 (44.84)	
Previous smoker	529 (16.40)	6349 (17.13)		482 (16.25)	545 (18.37)	
Current smoker	323 (10.02)	4055 (10.94)		297 (10.01)	295 (9.95)	
Unknown	857 (26.57)	9642 (26.01)		799 (26.94)	796 (26.84)	
Number of classes of GLAs initiated	1.26 ± 0.57	1.11 ± 0.35	< 0.001	1.25 ± 0.57	1.27 ± 0.58	0.269
**Index variables and move number of classes of GLAs**						
Body mass index^a^			< 0.001			0.991
Underweight/normal	128 (3.97)	4063 (10.96)		126 (4.25)	124 (4.18)	
Overweight	463 (14.36)	9447 (25.48)		438 (14.77)	425 (14.33)	
Obese class I	859 (26.64)	9735 (26.26)		811 (27.34)	828 (27.92)	
Obese class II	705 (21.86)	5826 (15.71)		638 (21.51)	627 (21.14)	
Obese class III	861 (26.70)	5350 (14.43)		754 (25.42)	760 (25.62)	
Unknown	209 (6.48)	2653 (7.16)		199 (6.71)	202 (6.81)	
High blood pressure (DBP ≥ 90 mmHg or SBP ≥ 140 mmHg)	963 (29.86)	11,751 (31.70)	0.031	883 (29.77)	871 (29.37)	0.733
**Pre-period general health**						
Charlson comorbidity index	1.53 ± 1.37	1.36 ± 1.46	< 0.001	1.50 ± 1.37	1.51 ± 1.48	0.777
Diabetes complications severity index	0.63 ± 1.08	0.61 ± 1.16	0.608	0.62 ± 1.08	0.62 ± 1.12	0.934
**Pre-period comorbidities**						
Anxiety	227 (7.04)	2316 (6.25)	0.076	199 (6.71)	197 (6.64)	0.917
Depression	370 (11.47)	3101 (8.36)	< 0.001	323 (10.89)	345 (11.63)	0.366
**Pre-period GLA use**						
Alpha-glucosidase Inhibitor	18 (0.56)	23 (0.06)	<0.001	12 (0.40)	13 (0.44)	0.841
Amylin	3 (0.09)	6 (0.02)	0.005	3 (0.10)	5 (0.17)	0.479
Basal insulin	488 (15.13)	509 (1.37)	< 0.001	360 (12.14)	323 (10.89)	0.132
Bolus insulin	235 (7.29)	189 (0.51)	< 0.001	157 (5.29)	120 (4.05)	0.023
DPP-4 inhibitor	401 (12.43)	466 (1.26)	< 0.001	300 (10.11)	286 (9.64)	0.542
Meglitinide	10 (0.31)	19 (0.05)	< 0.001	8 (0.27)	7 (0.24)	0.796
Metformin	825 (25.58)	1090 (2.94)	< 0.001	636 (21.44)	630 (21.24)	0.849
Pre-mixed insulin	75 (2.33)	94 (0.25)	< 0.001	54 (1.82)	51 (1.72)	0.768
Oral fixed combination	269 (8.34)	317 (0.86)	< 0.001	197 (6.64)	179 (6.04)	0.338
SGLT2 inhibitor	169 (5.2)	0 (0.00)	< 0.001	161 (5.4)	0 (0.00)	< 0.001
Sulfonylurea	306 (9.49)	537 (1.45)	< 0.001	243 (8.19)	242 (8.16)	0.962
Thiazolidinediones	124 (3.84)	196 (0.53)	< 0.001	101 (3.41)	96 (3.24)	0.717
**Pre-period other medication use**						
ACE-inhibitor	469 (14.54)	4061 (10.95)	< 0.001	411 (13.86)	391 (13.18)	0.448
Angiotensin receptor blocker	219 (6.79)	1939 (5.23)	< 0.001	189 (6.37)	189 (6.37)	1.000
Hypertension fixed-dose combination Therapy	295 (9.15)	2571 (6.93)	< 0.001	256 (8.63)	271 (9.14)	0.494
Non-steroidal anti-inflammatory drugs	415 (12.87)	4259 (11.49)	0.019	360 (12.14)	363 (12.24)	0.905
Aspirin	154 (4.78)	1379 (3.72)	0.003	124 (4.18)	121 (4.08)	0.845
Statin	859 (26.64)	6876 (18.55)	< 0.001	747 (25.19)	748 (25.22)	0.976
Ezetimibe	65 (2.02)	381 (1.03)	< 0.001	56 (1.89)	59 (1.99)	0.778
Number of classes of anti-hypertensives	0.58 ± 0.92	0.49 ± 0.85	< 0.001	1 ± 0.91	1 ± 0.89	0.488
**Pre-period visits to specialists**						
Endocrinologist	1.18 ± 2.79	0.41 ± 1.62	< 0.001	1.09 ± 2.61	1.11 ± 2.89	0.756
Cardiologist	0.10 ± 0.94	0.09 ± 0.91	0.359	0.10 ± 0.93	0.11 ± 1.04	0.703
**Index laboratory results**						
A1c	8.47 ± 1.86	7.77 ± 1.78	< 0.001	8.40 ± 1.86	8.43 ± 1.88	0.579
eGFR	82.69 ± 23.91	78.54 ± 23.40	< 0.001	82.22 ± 23.93	82.08 ± 23.77	0.823
eGFR category (in mL/min/1.73 m^2^)			< 0.001			0.644
≥ 90	1425 (44.19)	13,081 (35.28)		1282 (43.22)	1236 (41.67)	
< 90 and ≥ 60	1183 (36.68)	15,851 (42.87)		1104 (37.22)	1157 (39.01)	
< 60 and ≥ 45	383 (11.88)	4731 (12.76)		358 (12.07)	347 (11.70)	
< 45 and ≥ 30	179 (5.55)	2429 (6.55)		170 (5.73)	174 (5.87)	
< 30 and ≥ 15	48 (1.49)	795 (2.14)		45 (1.52)	47 (1.58)	
< 15	7 (0.22)	187 (0.50)		7 (0.24)	5 (0.17)	

Values in table are presented as the mean ± standard deviation or as a number of patients with the percentage in parenthesis, as appropriate

*ACE* Angiotensin-converting enzyme,* A1c* glycated hemoglobin,* DBP* diastolic blood pressure,* DDP-4* dipeptidyl peptidase 4,* eGFR*, estimated glomerular filtration rate,* GLA* glucose-lowering agent,* GLP-1 RA* glucagon-like peptide-1 receptor agonist,* SBP* systolic blood pressure,* SGLT2* sodium–glucose cotransporter 2

^a^Body mass index classifications based upon World Health Organization ( http://apps.who.int/bmi/index.jsp?introPage=intro_3.html)

Matching resulted in a successful match rate of 91.97%, with 2966 patients who initiated therapy with a GLP-1 RA and 2966 controls who initiated therapy with another class of other GLA. As Table 1 illustrates, the matching resulted in removing most of the differences between the two cohorts. However, post-matching there still remained a statistically significant difference in the percentage of patients who were prescribed bolus insulin in the pre-period, with patients who initiated therapy with a GLP-1 RA more likely to have received such a prescription compared to controls (5.29 vs. 4.05%; *P* = 0.023). In the matched cohort, patients who initiated therapy with a GLP-1 RA were on average 58.8 years old, and 52.49% were female, while 56.61% were identified as white and 72.99% were identified as non-Hispanic.

While Table 1 presents the descriptive statistics pre- and post-matching, Table 2 presents the distribution of patients prior to matching based upon eGFR categories. As Table 2 illustrates, there were significant differences in the class of drug initiated across eGFR categories (*P* < 0.0001), with the proportions of patients starting therapy with a GLP-1 RA tending to decrease with lower eGFR category. For example, the proportions of patients who initiated therapy with a GLP-1 RA were 7.5% of patients with an eGFR of < 60 and ≥ 45 mL/min/1.73 m^2^, 6.9% of patients with an eGFR of < 45 and ≥ 30 mL/min/1.73 m^2^, 5.7% of patients with an eGFR of < 30 and ≥ 15 mL/min/1.73 m^2^, and 3.6% of patients with an eGFR of < 15 mL/min/1.73 m^2^. Patients who initiated therapy with other GLAs were generally less likely to be treated with metformin or a SGLT2 inhibitor and more likely to be treated with insulin for lower eGFR categories. Overall, 2.6% of patients had an eGFR of < 30 mL/min/1.73 m^2^ and 94.7% of these patients (2.4% of the overall population) initiated therapy on a medication other than GLP-1 RA. Consistent with these results, the mean baseline eGFR prior to matching was significantly higher for patients who initiated a GLP-1 Ra compared to those who initiated therapy with an alternative GLA (82.69 vs. 78.54 mL/min/1.73 m^2^; *P* < 0.001).

**Table 2:** The distribution of patients by estimated glomerular filtration status based upon initiation of therapy with a glucagon-like peptide-1 receptor agonist therapy or any other glucose-lowering agent

Patient characteristics	eGFR (mL/min/1.73 m^2^)					
	≥ 90	< 90 and ≥ 60	< 60 and ≥ 45	< 45 and ≥ 30	< 30 and ≥ 15	< 15
Sample size	14,506 (36.0)	17,034 (42.3)	5114 (12.7)	2608 (6.5)	843 (2.1)	194 (0.5)
**Index therapy** ^a^						
GLP-1 RA	1425 (9.8)	1183 (6.9)	383 (7.5)	179 (6.9)	48 (5.7)	7 (3.6)
Other GLA	13,081 (90.2)	15,851 (93.1)	4731 (92.5)	2429 (93.1)	795 (94.3)	187 (96.4)
**Other GLA—by index class(es)**						
Basal Insulin	815 (5.6)	1042 (6.1)	443 (8.7)	391 (15.0)	179 (21.2)	59 (30.4)
Basal insulin + bolus insulin	–^b^	–^b^	110 (2.2)	80 (3.1)	47 (5.6)	19 (9.8)
Bolus insulin	279 (1.9)	381 (2.2)	194 (3.8)	175 (6.7)	72 (8.5)	23 (11.9)
DPP-4	963 (6.6)	1331 (7.8)	618 (12.1)	472 (18.1)	149 (17.7)	25 (12.9)
Metformin	6686 (46.1)	8079 (47.4)	1738 (34.0)	499 (19.1)	39 (4.6)	4 (2.1)
Metformin + sulfonylurea	424 (2.9)	396 (2.3)	101 (2.0)	–^b^	–^b^	–^b^
Oral fixed combination	1135 (7.8)	1182 (6.9)	264 (5.2)	75 (2.9)	–^b^	–^b^
Premix insulin	–^b^	–^b^	–^b^	55 (2.1)	37 (4.4)	8 (4.1)
Sulfonylurea	978 (6.7)	1408 (8.3)	624 (12.2)	435 (16.7)	164 (19.5)	36 (18.6)
SGLT2 inhibitor	479 (3.3)	487 (2.9)	105 (2.1)	–^b^	–^b^	–^b^
Thiazolinedione	–^b^	–^b^	136 (2.7)	82 (3.1)	24 (2.8)	–^b^
Other	1322 (9.1)	1545 (9.1)	398 (7.8)	165 (6.3)	84 (10.0)	13 (6.7)

Values in table are presented as the number of patients with the percentage given in parenthesis

^a^Chi square test examining difference in distribution of rental impairment between patients who initiate on GLP-1 RA therapy and those who initiate on an alternative GLA was statistically significant (*P* < 0.001)

^b^Indicates that < 2% of patients received this index class of therapy

**Fig. 2:**
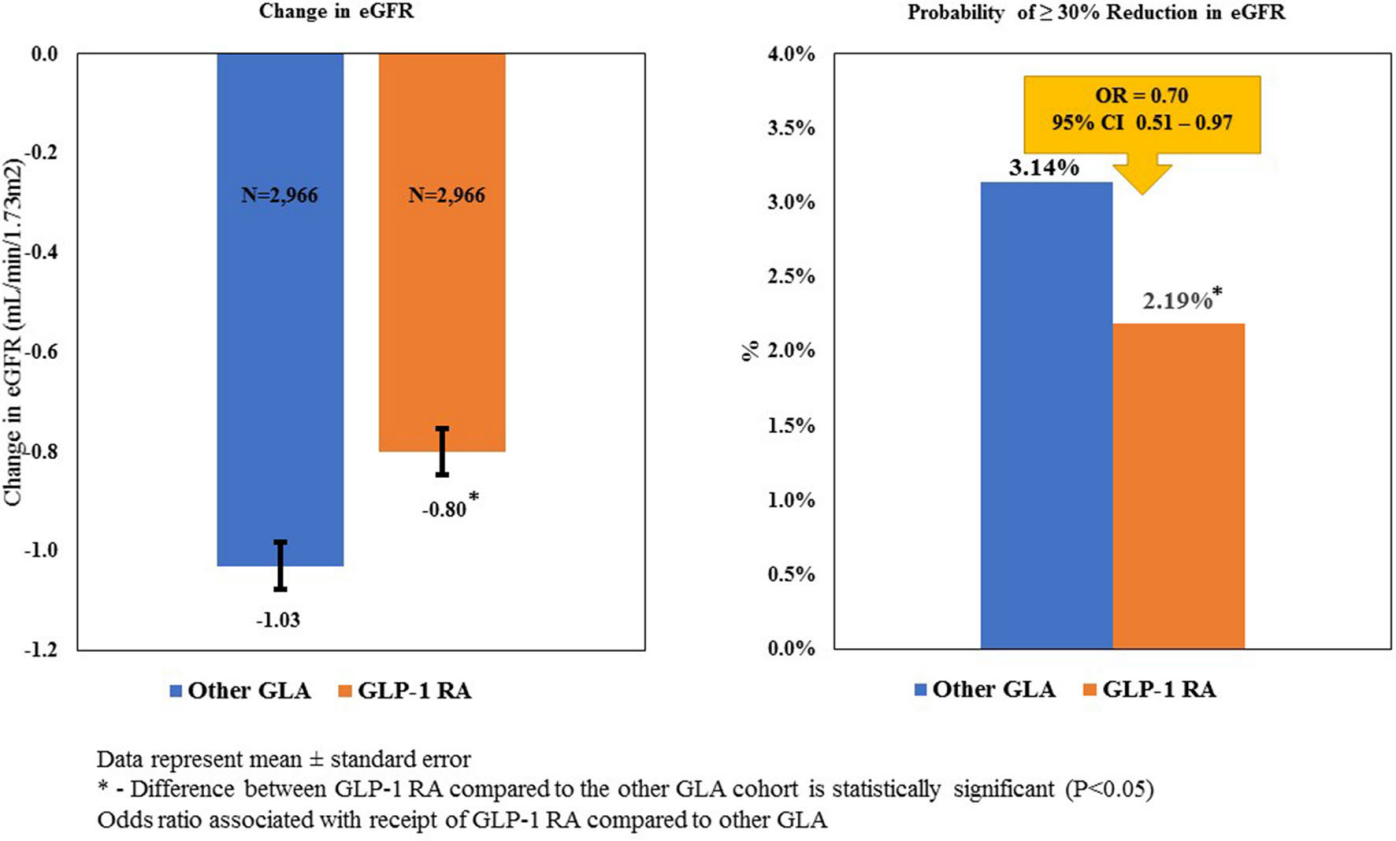
Kidney function of GLP-1 RAs compared to GLAs over time.* CI* Confidence interval,* eGFR* estimated glomerular filtration rate,* OR* odds ratio

Figure 2 illustrates the results of the multivariable analyses of the matched sample and reveals that patients who initiated therapy with a GLP-1 RA, compared to those who initiated therapy with an alternative GLA, had a statistically significantly smaller reduction in eGFR in the 1 year after therapy initiation (− 0.80 vs. − 1.03 mL/min/1.73 m^2^; *P* < 0.001). In addition, logistic regressions were estimated to examine the probability of patients having a ≥ 30% decrease in eGFR over the 1-year post-period. The results indicate that an estimated 2.19% of patients who initiated therapy on a GLP-1 RA had a reduction in eGFR of ≥ 30% compared to 3.14% of patients who initiated therapy with an alternative GLA (*P* < 0.001). The logistic regression results confirm this finding, with patients who initiated therapy with a GLP-1 RA found to be 30% less likely to have such a reduction over the 1-year post-period (OR 0.70, 95% CI 0.51–0.97).

**Fig. 3:**
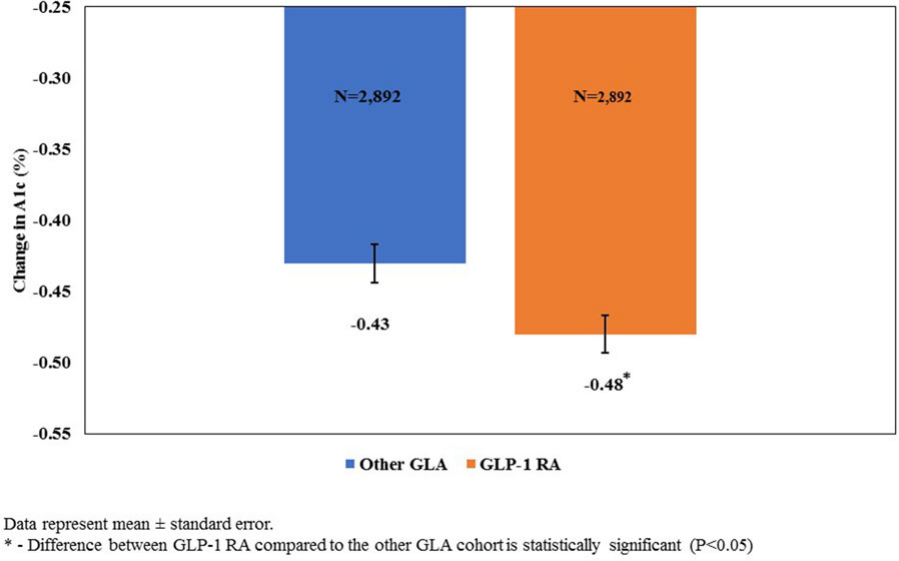
Reduction in glycated hemoglobin (*A1c*) with GLP-1 RA compared to other GLAs over time

In addition to examining eGFR, we also assessed A1c using a matched cohort of patients who had both an index and a post-period A1c score. There were 3158 patients who initiated therapy with a GLP-1 RA and 35,028 patients who initiated therapy with an alternative GLA who received at least one A1c test on or before the index date and another A1c test after the index date. Matching for this group resulted in a successful matched rate of 91.6%, with 2892 patients who initiated therapy with a GLP-1 RA and 2892 controls who initiated therapy with other GLAs. Given the similarity in sample sizes between this group of patients and the patients who received at least two serum creatinine tests, descriptive statistics pre- and post-matching are not presented. As with the eGFR cohort, after matching, the difference in the bolus insulin use prior to the index date remained statistically significant, with patients who initiated therapy with a GLP-1 RA significantly more likely to have received such a prescription (5.15 vs. 3.98%; *P* = 0.032). Results from the multivariable analyses conducted on the matched cohort (Fig. 3) revealed that patients who initiated therapy with a GLP-1 RA had a significantly larger reduction in A1c over the 1-year post-period compared to patients who initiated therapy with other GLAs (− 0.48 vs. − 0.43; *P* = 0.006). Multivariable analysis for the subset of individuals with an index eGFR of < 60 mL/min/1.73 m^2^ (*N* = 1067) revealed an even larger difference in A1c reduction for patients who initiated GLP-1 RA therapy compared to other GLA (− 0.41 vs − 0.22; *P* < 0.0001).

As a test of the sensitivity of the results, all analyses were conducted without matching of cohorts. There were no qualitative differences based upon these re-analyses. However, estimated differences in changes in eGFR and A1c were more pronounced in models without matching, where there was a mean difference of 0.36 mL/min/1.73 m^2^ in eGFR (− 0.83 vs − 1.19; *P* < 0.001) and a − 0.17% difference in A1c (− 0.50 vs. − 0.33; *P* < 0.001) associated with GLP-1 RA use compared to the use of other GLAS. The estimated likelihood of a ≥ 30% reduction in eGFR over the post-period was smaller (OR 0.77, 95% CI 0.59–0.99). Analyses were also performed with the inclusion of a variable that accounts for differences in timing between index and post-period measurements of eGFR and A1c. These analyses did not have any effect on the estimates of eGFR, although there was a slightly larger difference in A1c when patients who initiated therapy with a GLP-1 RA were compared to those who initiated therapy with other GLAs (− 0.46 vs − 0.40; *P* = 0.003).

## Discussion

Therapy with GLP-1 RA is an established treatment option for patients with T2D and has been shown to both improve glycemic control and be associated with weight loss without increasing the risk of hypoglycemia [24]. However, due to the lack of controlled studies and label restrictions [12, 25] there is limited use of this class in patients with moderate and severe renal impairment. This study found a decreasing use of GLP-1 RAs as renal impairment became more severe, with 5.7% of patients with an eGFR of < 30 and ≥ 15 mL/min/1.73 m^2^ and 3.6% of patients with an eGFR of < 15 mL/min/1.73 m^2^ initiating therapy with a GLP-1 RA. In the overall cohort, only 0.14% of patients had an eGFR of < 30 mL/min/1.73 m^2^ and initiated therapy with a GLP-1 RA, while 2.4% of patients had an eGFR of < 30 mL/min/1.73 m^2^ and initiated therapy with another GLA.

Multivariable analyses examined patient glycemic control over the 1-year post-period. The results of this study are consistent with those of clinical trials which illustrated that GLP-1 RAs are associated with reductions in A1c for patients with T2D [26–28], as well as for patients with T2D and moderate renal impairment [4]. The findings in this study are also consistent with those of retrospective, non-randomized studies that have illustrated the effectiveness of this class of drugs in real-world settings [29–31]. For example, a retrospective analysis of an electronic medical records database found that GLP-1 RA therapy, used with or without insulin, was associated with significant improvements in glycemic control [31]. Furthermore, the results are also in concert with analyses of claims databases which have shown that GLP-1 RAs are associated with greater improvement in glycemic outcomes relative to dipeptidyl peptidase-4 inhibitors [32, 33] and insulin glargine [34].

The smaller decline in eGFR and the lower likelihood of having a ≥ 30% reduction in eGFR associated with GLP-1 RA use compared to other GLAs found in this study generally complements the results of previous study types which have examined GLP-1 RA use and kidney function. For example, animal studies have shown that GLP-1 RA use is associated with natriuresis and diureses [35, 36] and that these agents also reduce risk factors of diabetic nephropathy, by reducing urine albumin levels [37] and inhibiting the development of hypertension [38]. Clinical trials have also illustrated the safety and potential benefits associated with use of GLP-1 RAs. For example, one clinical trial that compared GLP-1 RAs to placebo found no increased risk of acute renal failure associated with GLP-1 RA use [10], and another trial found similar rates of renal disorder adverse events among the two groups of patients [39]. In addition to their safety profile, the studies have also shown better patient outcomes associated with the use of GLP-1 RAs. For example, the SUSTAIN-6 study found semaglutide patients were significantly less likely to have new or worsening nephropathy [10] and the LEADER study showed a greater benefit of liraglutide with respect to cardiovascular outcomes in patients with an eGFR of < 60 ml/min/1.73 m^2^ [9]. The LEADER study also showed that use of liraglutide was associated with lower rates in the development and progression of diabetic kidney disease relative to placebo [40]. The AWARD-7 study, which compared dulaglutide and insulin glargine use, both combined with insulin lispro, in patients with T2D and stage 3 or 4 CKD found that the use of dulaglutide was associated with less eGFR decline at 26 weeks [41].

The findings reported herein must be interpreted within the context of the limitations of the study. First, a large percentage of T2D patients did not have recorded serum creatinine tests, suggesting that the results may not be generalizable to all patients. Second, the use of EHR data does not allow for confirmation that patients were filling and taking their prescribed medications. In addition, given the retrospective nature of the database, laboratory test results were not conducted at uniform time intervals for all patients. However, a sensitivity analysis revealed that the timing of these tests had no significant impact on the results. Third, the study focused exclusively on GLP-1 RAs as a class of medication and did not examine any potential differences among individual drugs. In addition, the study could not examine any potential impact of differences in GLP-1 RA doses on patient outcomes. Finally, it should be noted that the analysis focused on statistical significance, and practitioners should consider whether there are clinically important differences for the patients they treat. Specifically, the analysis focuses on whether there are statistical differences in outcomes and does not examine whether such differences represent meaningful changes to patients.

## Conclusions

Consistent with FDA labeling, this study found that the use of GLP-1 RAs was very limited in patients with a higher degree of renal impairment. However, multivariable analyses which controlled for a wide range of factors, including index eGFR, found that the initiation of therapy on GLP-1 RA was associated with a smaller decline in eGFR and larger reductions in A1c relative to the initiation of therapy on alternative classes of GLAs. Although more research may be needed for this population and individual GLP-1 RA therapies may differ in their respective risk–benefit ratio in patients with renal impairment, the findings of this study suggests that this class of drugs may be underutilized.

## References

[1] Neal B, Perkovic V, Matthews DR, Mahaffey KW, Fulcher G, Meininger G (2017). Rationale, design and baseline characteristics of the CANagliflozin cardioVascular Assessment Study-Renal (CANVAS-R): a randomized, placebo-controlled trial. Diabetes Obes Metab.

[2] U.S. Renal Data System. USRDS 2013 annual data report; atlas of chronic kidney disease and end-stage renal disease in the United States. National Institutes of Health, National Institute of Diabetes and Digestive and Kidney Disease [Internet], Bethesda, MD; 2014. Available from: https://www.usrds.org/atlas13.aspx.

[3] Pálsson R, Patel UD (2014). Cardiovascular complications of diabetic kidney disease. Adv Chronic Kidney Dis.

[4] Davies MJ, Bain SC, Atkin SL, Rossing P, Scott D, Shamkhalova MS (2016). Efficacy and safety of liraglutide versus placebo as add-on to glucose-lowering therapy in patients with type 2 diabetes and moderate renal impairment (LIRA-RENAL): a randomized clinical trial. Diabetes Care.

[5] Idorn T, Knop FK, Jørgensen MB, Jensen T, Resuli M, Hansen PM (2016). Safety and efficacy of liraglutide in patients with type 2 diabetes and end-stage renal disease: an investigator-initiated, placebo-controlled, double-blind, parallel-group, randomized trial. Diabetes Care.

[6] Alsahli M, Gerich JE (2015). Hypoglycemia in patients with diabetes and renal disease. J Clin Med.

[7] Park CW, Kim HW, Ko SH, Lim JH, Ryu GR, Chung HW (2007). Long-term treatment of glucagon-like peptide-1 analog exendin-4 ameliorates diabetic nephropathy through improving metabolic anomalies in db/db mice. J Am Soc Nephrol.

[8] Hendarto H, Inoguchi T, Maeda Y, Ikeda N, Zheng J, Takei R (2012). GLP-1 analog liraglutide protects against oxidative stress and albuminuria in streptozotocin-induced diabetic rats via protein kinase A-mediated inhibition of renal NAD(P)H oxidases. Metabolism.

[9] Marso SP, Daniels GH, Brown-Frandsen K, Kristensen P, Mann JFE, Nauck MA (2016). Liraglutide and cardiovascular outcomes in type 2 diabetes. N Engl J Med.

[10] Marso SP, Bain SC, Consoli A, Eliaschewitz FG, Jódar E, Leiter LA (2016). Semaglutide and cardiovascular outcomes in patients with type 2 diabetes. N Engl J Med.

[11] Kalra S (2016). Follow the LEADER—liraglutide effect and action in diabetes: evaluation of cardiovascular outcome results trial. Diabetes Ther.

[12] AstraZeneca Pharmaceuticals. Byetta (exenatide) injection. Highlights of prescribing information. Revised February 2015. [Internet]. Available from: https://www.azpicentral.com/byetta/pi_byetta.pdf#page=1. Accessed 22 June 2017.

[13] U.S. Drug and Food Administration (FDA). Adlyxin (lixisenatide) injection. Highlights of prescribing information. Revised July, 2016 [Internet]. Available from: https://www.accessdata.fda.gov/drugsatfda_docs/nda/2016/208471Orig1s000lbl.pdf. Accessed 26 Sept 2017.

[14] Marcus JR. A web-based electronic health record system for national surveillance. Online J Public Health Inform. [Internet]. 2013:5. Available from: http://www.ncbi.nlm.nih.gov/pmc/articles/PMC3692859/. Accessed 19 Mar 2017.

[15] Klompas M, Eggleston E, McVetta J, Lazarus R, Li L, Platt R (2013). Automated detection and classification of type 1 versus type 2 diabetes using electronic health record data. Diabetes Care.

[16] Levey AS, Stevens LA, Schmid CH, Zhang YL, Castro AF, Feldman HI (2009). A new equation to estimate glomerular filtration rate. Ann Intern Med.

[17] The Renal Association. CKD stages [Internet]. Available from: http://www.renal.org/information-resources/the-uk-eckd-guide/ckd-stages#sthash.s6SpVT7b.K0UCX4l0.dpbs. Accessed 17 Aug 2016.

[18] Rosenbaum PR, Rubin DB (1985). Constructing a control group using multivariate matched sampling methods that incorporate the propensity score. Am Stat.

[19] Deyo RA, Cherkin DC, Ciol MA (1992). Adapting a clinical comorbidity index for use with ICD-9-CM administrative databases. J Clin Epidemiol.

[20] Quan H, Sundararajan V, Halfon P, Fong A, Burnand B, Luthi J-C (2005). Coding algorithms for defining comorbidities in ICD-9-CM and ICD-10 administrative data. Med Care.

[21] Chang H-Y, Weiner JP, Richards TM, Bleich SN, Segal JB (2012). Validating the adapted diabetes complications severity index in claims data. Am J Manag Care.

[22] Grigsby AB, Anderson RJ, Freedland KE, Clouse RE, Lustman PJ (2002). Prevalence of anxiety in adults with diabetes: a systematic review. J Psychosom Res.

[23] Andreoulakis E, Hyphantis T, Kandylis D, Iacovides A (2012). Depression in diabetes mellitus: a comprehensive review. Hippokratia.

[24] Vergès B, Bonnard C, Renard E (2011). Beyond glucose lowering: glucagon-like peptide-1 receptor agonists, body weight and the cardiovascular system. Diabetes Metab.

[25] FDA. Victoza (liraglutide [rDNA origin] injection). Highlights of prescribing information [Internet]. Available from: https://www.accessdata.fda.gov/drugsatfda_docs/label/2010/022341lbl.pdf. Accessed 19 Mar 2017.

[26] Buse JB, Henry RR, Han J, Kim DD, Fineman MS, Baron AD (2004). Effects of exenatide (exendin-4) on glycemic control over 30 weeks in sulfonylurea-treated patients with type 2 diabetes. Diabetes Care.

[27] Buse JB, Nauck M, Forst T, Sheu WH-H, Shenouda SK, Heilmann CR (2013). Exenatide once weekly versus liraglutide once daily in patients with type 2 diabetes (DURATION-6): a randomised, open-label study. Lancet.

[28] Nauck M, Frid A, Hermansen K, Shah NS, Tankova T, Mitha IH (2009). Efficacy and safety comparison of liraglutide, glimepiride, and placebo, all in combination with metformin, in type 2 diabetes: the LEAD (liraglutide effect and action in diabetes)-2 study. Diabetes Care.

[29] Durden E, Lenhart G, Lopez-Gonzalez L, Hammer M, Langer J (2016). Predictors of glycemic control and diabetes-related costs among type 2 diabetes patients initiating therapy with liraglutide in the United States. J Med Econ.

[30] Heymann A, Maor Y, Goldstein I, Todorova L, Schertz-Sternberg P, Karasik A (2014). Efficacy of liraglutide in a real-life cohort. Diabetes Ther.

[31] Singhal M, Unni S, Schauerhamer M, Nguyen H, Hurd J, McAdam-Marx C (2017). Real-world glycemic control from glp-1ra therapy with and without concurrent insulin in patients with type 2 diabetes. J Manag Care Spec Pharm.

[32] Lee WC, Dekoven M, Bouchard J, Massoudi M, Langer J (2014). Improved real-world glycaemic outcomes with liraglutide versus other incretin-based therapies in type 2 diabetes. Diabetes Obes Metab.

[33] Li Q, Chitnis A, Hammer M, Langer J (2014). Real-world clinical and economic outcomes of liraglutide versus sitagliptin in patients with type 2 diabetes mellitus in the United States. Diabetes Ther.

[34] Thayer S, Wei W, Buysman E, Brekke L, Crown W, Grabner M, et al. The INITIATOR study: pilot data on real-world clinical and economic outcomes in US patients with type 2 diabetes initiating injectable therapy. Adv Ther. 2013;30(12):1128–1140. 10.1007/s12325-013-0074-8 PMC3898354 24293131

[35] Kim M, Platt MJ, Shibasaki T, Quaggin SE, Backx PH, Seino S (2013). GLP-1 receptor activation and Epac2 link atrial natriuretic peptide secretion to control of blood pressure. Nat Med.

[36] Rieg T, Gerasimova M, Murray F, Masuda T, Tang T, Rose M (2012). Natriuretic effect by exendin-4, but not the DPP-4 inhibitor alogliptin, is mediated via the GLP-1 receptor and preserved in obese type 2 diabetic mice. Am J Physiol Renal Physiol.

[37] Cummings BP, Stanhope KL, Graham JL, Baskin DG, Griffen SC, Nilsson C (2010). Chronic administration of the glucagon-like peptide-1 analog, liraglutide, delays the onset of diabetes and lowers triglycerides in UCD-T2DM rats. Diabetes.

[38] Liu Q, Adams L, Broyde A, Fernandez R, Baron AD, Parkes DG (2010). The exenatide analogue AC3174 attenuates hypertension, insulin resistance, and renal dysfunction in Dahl salt-sensitive rats. Cardiovasc Diabetol.

[39] Tuttle KR, McKinney TD, Davidson JA, Anglin G, Harper KD, Botros FT (2017). Effects of once-weekly dulaglutide on kidney function in patients with type 2 diabetes in phase II and III clinical trials. Diabetes Obes Metab.

[40] Mann JFE, Ørsted DD, Brown-Frandsen K, Marso SP, Poulter NR, Rasmussen S (2017). Liraglutide and renal outcomes in type 2 diabetes. N Engl J Med.

[41] Tuttle K, Lakshmanan M, Gross J, Rayner B, Busch R, Zimmermann A (2017). Dulaglutide versus glargine, both combined with lispro, mitigated eGFR decline in people with type 2 diabetes and moderate to severe chronic kidney disease (AWARD-7). Diabetes.

